# Feel the force: Biomechanical homeostasis of the cardiovascular system

**DOI:** 10.1515/jtim-2026-0015

**Published:** 2026-02-13

**Authors:** Quanyou Shi, Ming Xu, Chi Zhu, Guoping Shi

**Affiliations:** Department of Cardiology and Institute of Vascular Medicine, Peking University Third Hospital, State Key Laboratory of Vascular Homeostasis and Remodeling, NHC Key Laboratory of Cardiovascular Molecular Biology and Regulatory Peptides, Beijing, China; Research Unit of Medical Science Research Management/Basic and Clinical Research of Metabolic Cardiovascular Diseases, Chinese Academy of Medical Sciences, Beijing, China; School of Mechanics and Engineering Science, Peking University, Beijing, China; Zhongda Hospital, Advanced Institute for Life and Health, School of Medicine, Southeast University, Nanjing, Jiangsu Province, China; Department of Medicine, Brigham and Women's Hospital and Harvard Medical School, Boston, MA, USA

## Introduction

Both chronic and acute cardiovascular diseases (CVDs) involve dysregulation of biomechanical homeostasis, including hemodynamic forces and structural mechanics. Hemodynamic forces are flow-derived stresses, including shear stress, flow pressure, and impedance that regulate blood cell dynamics and endothelial functions. In contrast, structural mechanics refers to tissue-level loads and properties, such as wall stress, strain, and stiffness that govern myocardial and vascular wall remodeling, yet have been largely overlooked relative to hemodynamic forces. These two domains, hemodynamic forces and structural mechanics, are tightly coupled and should be interpreted together rather than independently.

**Figure 1:**
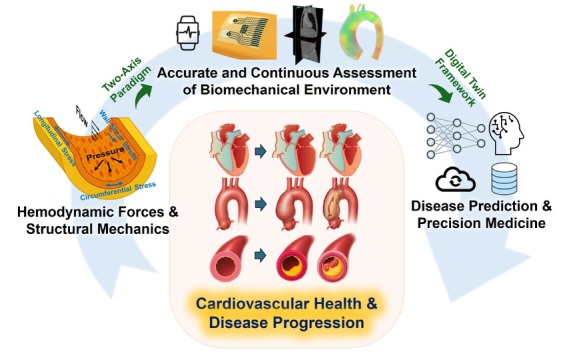
“Feel the force” in cardiovascular health management. Schematic illustration of the proposed two-axis paradigm, in which hemodynamic forces and structural mechanics jointly define cardiovascular biomechanical homeostasis and synergistically regulate the progression of cardiovascular disease. Advances in medical imaging, wearables, computational simulation are leading to accurate and continuous measurement of biomechanica environment. These multimodal biomechanical data can be fused by artificial intelligence within the cardiovascular digital twin framework, which is capable of continuous cardiovascular monitoring and risk prediction, enabling precision medicine.

Long-standing deviation of either domain from physiological ranges drives maladaptive remodeling that contributes to chronic CVDs such as heart failure (HF), cardiomyopathy, aortic aneurysm, and atherosclerosis, while abrupt perturbation precipitates acute catastrophic events, including myocardial infarction, aortic dissection, and stroke. Precise assessment and continuous monitoring of force environments may reflect current cardiovascular health status and predict long-term and short-term future cardiovascular risks, enabling pre-emptive intervention. This perspective emphasizes the importance of both hemodynamic forces and structural mechanics in cardiovascular health and highlights the need for accurate, noninvasive, and continuous monitoring of the biomechanical environment and clinical translation towards biomechanics-informed and digital-twin-driven frameworks in disease risk prediction (Figure 1).

## Hemodynamic forces and structural mechanics in cardiovascular disease progression

Heart, aortic, and systemic vasculatures form a closed-loop circulatory system, in which changes in one node propagate throughout the network. Heart exhibits both passive stiffness and active contractile properties, governed by the Frank-Starling law, correlating to its diastolic and systolic functions. Heart acts like an engine that drives blood flow in the aorta and systemic vasculatures but is simultaneously modulated by arterial hemodynamics and venous return. Alterations in blood preload and afterload affect structural heart load differentially and induce distinctive cardiac remodeling (eccentric or concentric hypertrophy), leading to different HF phenotypes: HF with reduced ejection fraction (HFrEF) and HF with preserved ejection fraction (HFpEF).

Besides heart, biomechanical environment is also a key driver for aortic pathogenesis. On the hemodynamic side, wall shear stress (WSS), the tangential frictional force exerted by the blood flow on endothelial cells, is well-studied and shows close association with the progression of aortic diseases such as atherosclerosis. For example, the oscillatory and low WSS is atheroprone that fosters endothelial dysfunction and atherosclerotic plaque progression, while physiological, unidirectional WSS is atheroprotective. On the structural side, mechanical wall stress (MWS), the tensile and compressive loads within the aortic wall, has gained increasing attention in recent years as a determinant of aortic disease progression. High MWS is associated positively with aortic dissection, aneurysm progression, and atherosclerotic plaque rupture. At the cellular level, WSS regulates mainly the homeostasis of endothelial cells (*e.g*., barrier function, inflammation, mechano-signaling), while MWS modulates the switch of smooth muscle cells between the contractile and synthetic phenotypes.^[1]^ Perturbations of biomechanical forces can further propagate to subcellular organelles, including mitochondria, contributing to endothelial dysfunction.^[2]^

Importantly, WSS (hemodynamic) and MWS (structural) act synergistically rather than independently. Recent studies combining intravascular imaging and computational modeling quantified WSS and MWS simultaneously and demonstrated their joint role in atherosclerotic plaque development and rupture.^[3,4]^ Studies utilizing CTA imaging, histopathology and computational modeling showed that incorporation of structural mechanics and hemodynamics improved the understanding of cerebral aneurysm rupture.^[5]^ Future studies integrating WSS and MWS may provide insights of how hemodynamic forces and structural mechanics interplay and synergistically regulate aortic disease progression.

Given both the acute and chronic consequences of biomechanical forces, accurate and continuous monitoring is clinically essential, not only to assess current cardiovascular health status but also to provide early warnings for future cardiovascular events.

## Accurate and continuous noninvasive assessment of cardiovascular biomechanical environment

Accurate and noninvasive assessment of hemodynamic forces and, especially, structural mechanics has long been challenging, but recent progress in medical imaging is minimizing this gap. Techniques such as 4D flow magnetic resonance imaging (MRI), vector flow imaging, and 3D ultrafast ultrasound can now provide high-resolution, three-dimensional assessment of the hemodynamic environment, including flow velocity and WSS. On the other hand, innovations in ultrasound and MRI, such as ultra-high-frequency ultrasound, shear-wave elastography, and cardiac MRI, enabled increasingly detailed assessment of the structural mechanics, including myocardial strain and aortic wall stiffness.^[6]^

Beyond the clinical or bedside setting, continuous biomechanical monitoring in daily life is becoming feasible with the recent development of wearable technologies. Advances in material engineering and signal processing algorithms have led to wearable devices that are capable of continually sensing biomechanical environment. A wearable ultrasonic device attached to chest was reported to provide direct real-time cardiac function assessment.^[7]^ A fully integrated wearable ultrasonic-system-on-patch (USoP) demonstrated 12-hour autonomous monitoring of physiological signals, including central blood pressure, heart rate, and cardiac output.^[8]^ Wearable devices attached to radial and brachial arteries were reported to provide continuous cardiovascular monitoring and showed great translational capability.^[9]^ Such wearable devices generate high-frequency waveform data of body biomechanical environment, as a complementary modality to the currently established signals such as electrocardiogram (ECG) and photoplethysmogram (PPG).

In parallel, computational simulation, particularly fluid structure interaction (FSI), remains the gold standard in biomechanical research. FSI simultaneously resolves both blood flow dynamics (hemodynamics) and aortic wall deformation (structural mechanics), providing a comprehensive, coupled assessment of biomechanical environment that is difficult to obtain directly from the *in vivo* setting. However, traditional FSI solvers are computationally demanding and slow. Recent progress integrating artificial intelligence (AI) with numerical simulation achieved orders-of-magnitude acceleration without substantial loss of accuracy.^[10]^ Together, these technologies have substantially advanced our ability to depict noninvasively our biomechanical environment across spatial and temporal scales.

## Biomechanics-informed digital twins for cardiovascular monitoring and risk prediction

Despite these impressive advances, each tool has intrinsic limitations that hinder their broad clinical adoption. Medical imaging offers high-resolution biomechanical information but is constrained by its cost and accessibility. Wearable devices provide continuous monitoring but are restricted by sensor precision, battery life, and motion artifacts. Computation simulation depends heavily on patient anatomical data and boundary conditions. Given these complementary strengths and weaknesses of each tool, the vision of a cardiovascular digital twin, which is a personalized computational replica of individual patient, requires synergistic integration of these approaches. High-resolution imaging and FSI simulation can jointly establish a patient-specific biomechanical baseline model, which can be further updated with real-world dynamics by continuous wearable monitoring. AI, supported by edge computing and cloud computing, can fuse and interpret these multimodal inputs in real time. Such integrated framework of digital twin could ultimately achieve not only cardiovascular health monitoring, but also future disease risk prediction.

Recent studies illustrate this potential. A digital twin for abdominal aortic aneurysm (AAA) was built based on transmission line model that enabled personalized AAA monitoring through noninvasively measured tonometry waveform.^[11]^ Another study similarly showed great predictive capability of noninvasively measured carotid and femoral atrial pressure waveforms with a receiver operating characteristic area under the curve (ROC AUC) of 0.83 ± 0.04 for identification of AAA patients.^[12]^ Digital twin for pulmonary artery based on continuous noninvasive monitoring of hemodynamic forces could provide early identification of right ventricular worsening in HF patients.^[13]^ Despite these promising studies, current digital twins typically model isolated components, such as pulmonary artery, coronary artery, or abdominal aorta. Future research should aim to integrate these modules into an interconnected whole-system representation. Furthermore, the next generation of digital twins should incorporate additional data modalities, including electrophysiology and multi-omics data to enable multiscale calibration and true patient-specific prediction, advancing the vision of precision cardiovascular medicine. For example, ECG coupling enabled an electromechanical whole-heart digital model that reproduced pressure-volume dynamics of HF patients.^[14]^ Yet, digital twin framework still faces several barriers before it can be accepted broadly in clinical practice. These include challenges in multimodal data integration, need for standardized pipelines for model validation, and requirement for large-scale prospective clinical trials to demonstrate safety and clinical benefit. Practical constraints, such as computational cost, regulatory governance approval, data sharing and cybersecurity, also need to be systemically addressed to fully realize the vision of cardiovascular digital twin for precision medicine.^[15]^

## Conclusion

In this perspective, we propose a two-axis paradigm by combining hemodynamic forces (*e.g*., WSS) and structural mechanics (*e.g*., MWS) when assessing biomechanical environment and studying its role in cardiovascular health. Digital-twin frameworks—integrating computational modeling, medical imaging data, continuous wearables monitoring, and AI models—will enable continuous monitoring of patient-specific force profiles. Such integration will allow early detection of maladaptive remodeling, warn for future disease risk, and potentially provide virtual testing platforms for therapeutic interventions. Together, these advances may lay the foundation for precision and proactive cardiology in which clinicians can “feel the force”, so that the cardiovascular diseases are not merely treated after onset but also predicted, prevented, and personalized through the language of forces.

## References

[1] Davis MJ, Earley S, Li YS, Chien S (2023). Vascular mechanotransduction. Physiol Rev.

[2] Pang B, Dong G, Pang T, Sun X, Liu X, Nie Y (2024). Emerging insights into the pathogenesis and therapeutic strategies for vascular endothelial injury-associated diseases: focus on mitochondrial dysfunction. Angiogenesis.

[3] Tziotziou A, Hartman E, Korteland SA, van der Lugt A, van der Steen AFW, Daemen J (2023). Mechanical wall stress and wall shear stress are associated with atherosclerosis development in non-calcified coronary segments. Atherosclerosis.

[4] Wang L, Zhu Y, Zhao C, Maehara A, Lv R, Guo X (2025). Author Correction: Role of biomechanical factors in plaque rupture and erosion: insight from intravascular imaging based computational modeling. npj Cardiovasc Health.

[5] Alagan AK, Valeti C, Bolem S, Karve OS, Arvind KR, Rajalakshmi P (2025). Histopathology-based near-realistic arterial wall reconstruction of a patient-specific cerebral aneurysm for fluid-structure interaction studies. Comput Biol Med.

[6] Jiang Y, Li GY, Hu K, Ma S, Zheng Y, Jiang M (2025). Simultaneous imaging of bidirectional guided waves probes arterial mechanical anisotropy, blood pressure, and stress synchronously. Sci Adv.

[7] Hu H, Huang H, Li M, Gao X, Yin L, Qi R (2023). A wearable cardiac ultrasound imager. Nature.

[8] Lin M, Zhang Z, Gao X, Bian Y, Wu RS, Park G (2024). A fully integrated wearable ultrasound system to monitor deep tissues in moving subjects. Nat Biotechnol.

[9] Zhou S, Park G, Longardner K, Lin M, Qi B, Yang X (2025). Clinical validation of a wearable ultrasound sensor of blood pressure. Nat Biomed Eng.

[10] Tanade C, Khan NS, Rakestraw E, Ladd WD, Draeger EW, Randles A (2024). Establishing the longitudinal hemodynamic mapping framework for wearable-driven coronary digital twins. NPJ Digit Med.

[11] Kim D, Narayanan D, Sung SH, Cheng HM, Chen CH, Kim CS (2024). Transmission line model as a digital twin for abdominal aortic aneurysm patients. NPJ Digit Med.

[12] Yavarimanesh M, Cheng HM, Chen CH, Sung SH, Mahajan A, Chaer RA (2022). Abdominal aortic aneurysm monitoring via arterial waveform analysis: towards a convenient point-of-care device. NPJ Digit Med.

[13] Geddes JR, Jensen CW, Tanade C, Ghorbannia A, Fudim M, Patel MR (2025). Digital twins for noninvasively measuring predictive markers of right heart failure. NPJ Digit Med.

[14] Salvador M, Strocchi M, Regazzoni F, Augustin CM, Dede’ L, Niederer SA (2024). Whole-heart electromechanical simulations using Latent Neural Ordinary Differential Equations. NPJ Digit Med.

[15] Coorey G, Figtree GA, Fletcher DF, Snelson VJ, Vernon ST, Winlaw D (2022). The health digital twin to tackle cardiovascular disease-a review of an emerging interdisciplinary field. NPJ Digit Med.

